# Sex-specific associations between dietary nutrient intake and cardiometabolic biomarkers in older adults with diet-related chronic diseases

**DOI:** 10.3389/fnut.2026.1877635

**Published:** 2026-08-14

**Authors:** Svetlana Plyassovskaya, Olzhas Zhamantayev, Gulmira Zhanalina, Bakhyt Kosherova, Nurbek Yerdessov

**Affiliations:** 1 School of Public Health, Karaganda Medical University, Karaganda, Kazakhstan; 2 Department of Infectious Diseases and Phthisiology, Karaganda Medical University, Karaganda, Kazakhstan

**Keywords:** cardiometabolic risk, elderly, Kazakhstan, micronutrients, nutrition, type 2 diabetes

## Abstract

**Background:**

Older adults presenting with concurrent type 2 diabetes mellitus (T2DM), arterial hypertension (AH), and obesity face a considerable cardiometabolic challenge. There were no recent studies examining the association between their habitual nutrient consumption and circulating biomarkers in Central Asian populations. We aimed to examine sex-stratified associations between daily nutrient consumption and cardiometabolic biomarkers in community-dwelling older adults aged 60–90 years with concurrent T2DM, AH, and obesity in Central Kazakhstan.

**Methods:**

We enrolled 351 community-dwelling adults aged 60–90 years (80 men, 271 women) from four primary care facilities. Fasting glucose, total cholesterol, creatinine, urea, and triglycerides were assessed. Nutrient adequacy was expressed as percentage of Kazakhstani reference value. Spearman rank correlation, multiple linear regression, and binary logistic regression assessed dietary-biomarker relationships, stratified by sex.

**Results:**

We identified the widespread micronutrient inadequacies in both sexes. Median potassium intake reached 34.3% of the reference value in men and 32.9% in women; calcium 33.6 and 38.1%; vitamin C 22.3 and 30.5%; vitamin E 43.7 and 38.9%; thiamine 48.1 and 49.5%. Sodium intake exceeded the reference value in both sexes, more than twofold in men and close to twofold in women. In men, total fat and saturated fat intake correlated with creatinine (*r*s = 0.242 and 0.250, respectively; *p* < 0.05), and total fat also correlated with triglycerides (*r*s = 0.243, *p* = 0.030). BMI was the leading independent triglyceride predictor (*β* = 0.572, *p* < 0.01, *R*^2^ = 0.333). In women, most dietary–biomarker associations were absent, and calcium and riboflavin showed inverse correlations with triglycerides (*r*s = −0.237, *p* < 0.001 and *r*s = −0.164, *p* = 0.007).

**Conclusion:**

Older Kazakhstani adults with T2DM, AH, and obesity showed near-universal shortfalls in potassium, calcium, vitamins C and E, and thiamine alongside sodium excess. Dietary–biomarker associations were sex-specific, driven primarily by adiposity in men. Correcting these micronutrient gaps and reducing sodium intake are priorities for chronic disease management in this population.

## 1 Introduction

The proportion of older adults around the world keeps rising, with no signs of slowing down. By 2050, the global population aged 60 years and older is projected to more than double, from about 1 billion to 2.1 billion (1, 2). As people age, they face ongoing declines in organ function, changes in body composition, and a higher risk of chronic non-communicable diseases. In geriatric care, few patterns of multiple health problems are as important as the combination of T2DM, AH, and obesity, often called diet-related or alimentary-dependent diseases, since diet plays a key role in how these conditions start and develop (3, 4). Dietary macro- and micronutrients affect insulin sensitivity, lipid metabolism, blood pressure, and systemic inflammation through well-characterized biochemical pathways (5, 6), and meal-timing patterns such as intermittent fasting affect several of the same risk factors (7). For older adults, nutrition becomes even more complex because energy intake drops with age, nutrient absorption can get worse, medicines pile up, and limited finances might make it harder to get healthy food. Together, these issues can lead to getting too many macronutrients, but not enough micronutrients (8, 9). Protein inadequacy promotes sarcopenia (10, 11), calcium and vitamin D deficits accelerate bone loss (12), and low intake of antioxidant vitamins (particularly vitamins C and E) has been associated with impaired glycemic control and exacerbation of vascular endothelial dysfunction (13, 14).

The association between habitual dietary intake and cardiometabolic biomarkers (fasting glucose, total cholesterol, triglycerides, and markers of renal nitrogen metabolism) is not a fixed biological property. It is shaped by sex-related biology at multiple levels. Men and women differ in fat mass distribution, skeletal muscle mass, hormonal background, and renal handling of metabolic by-products (15, 16). These differences produce divergent dietary–biomarker associations across sexes. Postprandial lipemia is more pronounced in men than in pre-menopausal women under equivalent dietary fat loads, a difference that persists in attenuated form into older age groups (17). Women with a higher proportion of body fat tend to show an expanded plasma volume, a hemodilution effect that can weaken the observed association between dietary intake and circulating biomarker concentrations, most clearly documented for iron status and vitamin D (18). Most nutritional epidemiology studies conducted in older populations do not stratify their analyses by sex, and those that attempt to do so are commonly underpowered to detect sex-by-diet interaction effects (19).

In Kazakhstan and the wider Central Asian region, there is not much data on the nutritional status of older people with diet-related diseases in the era of nutrition transition. What we do know suggests that the traditional diet here is heavy on refined carbohydrates, animal fats, and salt, but low in fruits, vegetables, and fish. This kind of eating pattern likely raises cardiometabolic risk, but few detailed dietary studies link intake to laboratory biomarkers in this region (20, 21). The “Countrywide Integrated Noncommunicable Disease Intervention” (CINDI) program, which Kazakhstan used through its national health development plan called “Densaulyk,” gives a solid way to assess diet for age and sex comparisons across the population. Until now, no published sex-disaggregated analysis has examined how nutrients and biomarkers are linked in this context (22, 23).

This study addresses that gap. Using data from a cross-sectional sample of community-dwelling older adults in Central Kazakhstan, each with a confirmed alimentary-dependent disease, we examined associations between habitual nutrient intake and five cardiometabolic biomarkers: fasting glucose, total cholesterol, triglycerides, creatinine, and urea. We also characterized the prevalence of micronutrient inadequacy in both men and women, and assessed the relationship between dietary fat intake and triglyceride concentration across intake tertiles. Prior to analysis, we specified two directional hypotheses: first, that dietary–biomarker associations would be stronger in men than in women, and second, that in women, selected micronutrients would show an inverse association with serum lipid markers. Associations identified outside these pre-specified hypotheses are treated as exploratory observations and reported as such throughout.

## 2 Materials and methods

### 2.1 Study design and participants

This cross-sectional study took place within four primary care centers in Central Kazakhstan between March 2 and March 23, 2026. The participants were adults living in the community, aged 60–90, with a confirmed, non-insulin-treated T2DM diagnosis at the time of enrollment (with the disease lasting 3–10 years). They were also either overweight or obese (BMI ≥ 25 kg/m^2^) and had grade 1 or 2 AH, defined as office systolic blood pressure between 140 and 179 mmHg and/or diastolic blood pressure between 90 and 109 mmHg according to the 2023 European Society of Hypertension classification (24), with all these conditions confirmed through outpatient medical records. Participants were excluded if they had severe chronic conditions, including active malignancy, decompensated renal or hepatic failure, or advanced heart failure, a psychiatric disorder precluding informed consent, any acute illness in the 3 months preceding enrollment or concurrent participation in another dietary or metabolic intervention study. The Karaganda Medical University Bioethics Committee approved the study (protocol No.5, February 25, 2026), and every participant signed a written consent form before enrolling. The final group included 80 men (22.8%) and 271 women (77.2%).

Participants were divided into age groups following the classification used in prior work on this population (25): young-old (60–74 years) and old-old (75–90 years). The predominance of women in this sample parallels the sex distribution reported in that earlier study of the same population (25) and is consistent with the general tendency of women in this age group to use primary care more often than men with a comparable disease burden (26).

### 2.2 Dietary assessment and anthropometric measurements

Dietary intake was assessed by a single 24-h dietary recall administered at a morning clinic appointment, using the CINDI dietary assessment protocol endorsed by the WHO European Regional Office (22). The CINDI protocol also includes a food frequency questionnaire designed to characterize habitual food consumption patterns. Although this component was administered as part of the standardized assessment, it was not used for quantitative nutrient estimation in the present study. Accordingly, all macronutrient and micronutrient intake values reported in this manuscript were derived exclusively from the 24-h dietary recall. Interviewers used portion-size estimation aids and food composition tables approved by the National Sanitary-Epidemiological Control Committee of Kazakhstan. Participants were asked to report all foods and beverages consumed during the preceding 24 h. Dietary recalls were collected throughout the study period and scheduled across weekdays and weekends in an approximate 4:1 ratio, with national holidays excluded.

All participants underwent the same anthropometric assessment and dietary interview according to the study protocol. Daily intake of macronutrients (protein, total fat, carbohydrates, saturated fatty acids [SFA], mono- and disaccharides, starch), dietary fiber, minerals (sodium, potassium, calcium, magnesium, phosphorus, iron), and vitamins (retinol equivalents [RE], tocopherol equivalents [TE], thiamine [B1], riboflavin [B2], niacin equivalents [NE], and ascorbic acid [vitamin C]) was computed from the electronic database of the chemical composition of foods of Kazakhstan. Total energy intake (kcal/day) came from the macronutrient contributions. Nutrient adequacy was expressed as a percentage of the age- and sex-specific reference value, using the 2023 Methodological Guidelines from the Ministry of Health of the Republic of Kazakhstan and FAO/WHO reference values (27, 28). The reference values applied for sodium (1,300 mg/day for participants aged 60–74 years and 1,200 mg/day for those aged 75–90 years) reflect a physiological requirement level rather than the World Health Organization’s population-level ceiling of below 2000 mg/day for the general adult population. The two are not directly comparable, and this distinction is addressed further in Section 4.

Standing height was measured to the nearest 0.5 cm with a portable stadiometer, while participants were shoeless and relaxed. Weight and body composition were measured in the fasting state with the SC-330S TANITA segmental bioelectrical impedance analyzer (TANITA Corporation, Tokyo, Japan). BMI was computed as weight in kilograms divided by the square of height in meters.

### 2.3 Biochemical measurements

Fasting venous blood samples provided concentrations of glucose (mmol/L), total cholesterol (mmol/L), creatinine (μmol/L), urea (mmol/L), and triglycerides (mmol/L). All values were extracted from ambulatory medical records. Samples were collected at the same clinic visit as the dietary recall or, where the participant had an intervening scheduled blood draw within 14 days, from that draw. Values obtained more than 14 days from the dietary recall date were excluded. For prevalence estimation, the following clinical thresholds were applied: hyperglycemia at fasting glucose above 7.0 mmol/L, hypercholesterolemia at total cholesterol above 5.2 mmol/L, hypertriglyceridemia at triglycerides above 1.7 mmol/L, and elevated creatinine above 115 μmol/L in men and above 97 μmol/L in women, in line with conventional reference limits. Because values were drawn from routine records across four separate primary care laboratories rather than a single research laboratory, minor inter-laboratory variability in assay method cannot be excluded; this is addressed as a limitation in Section 4.

### 2.4 Statistical analysis

All analyses were performed in SPSS 27.0 (IBM Corp., Armonk, NY, USA). Normally distributed measures appear as mean ± SD, and non-normally distributed continuous data are reported as median with interquartile range (IQR). We compared continuous variables between sexes with Mann–Whitney *U* test, and categorical distributions with chi-square test. Spearman rank correlation coefficients (*r*s) with two-tailed *p*-values showed the associations between daily nutrient intake and each biomarker concentration. These analyses were done separately for men and women. Multiple linear regression with standardized predictors modeled serum triglyceride concentration within each sex, letting us directly compare effect sizes as standardized *β* coefficients. Total fat intake tertiles were defined separately for men and women, and Kruskal-Wallis tests checked if triglyceride and glucose levels changed across tertiles. Micronutrient inadequacy was defined as the proportion of participants with intake below 80% of the age- and sex-specific reference value, a threshold commonly used for geriatric nutrition screening (8, 29). Binary logistic regression with the same set of seven standardized predictors modeled the chance of hypertriglyceridemia (serum triglycerides > 1.7 mmol/L) in each sex. One male participant with missing BMI data was excluded from the sex-specific logistic regression models, yielding an analytic sample of 79 men for that analysis. For women, an extended model also included dietary calcium and riboflavin as predictors, since they correlated with serum triglycerides in bivariate analysis. Discriminative capacity was measured by the area under the receiver-operating characteristic curve (AUROC). Overall model fit was checked with the McFadden pseudo-R^2^ and the likelihood-ratio chi-square test. All tests were two-tailed at a = 0.05. These correlation analyses were exploratory. Given the large number of nutrient–biomarker pairs tested, no correction for multiple comparisons was applied. The resulting coefficients carry a non-trivial probability of including false-positive associations and should not be interpreted as independent confirmatory findings. Throughout the Results and Discussion, statistically significant correlations are presented as hypothesis-generating observations whose biological plausibility is considered, but whose directionality and magnitude require replication in studies with repeated dietary measures and prospective designs.

Triglyceride values did not have a normal distribution in both sexes (Shapiro–Wilk *p* < 0.001 on model residuals). As a sensitivity check, the multiple linear regression was repeated with natural-log-transformed triglycerides as the outcome. The pattern of associations held up well (Supplementary Table S1), and the binary logistic regression on hypertriglyceridemia served as a further, distributional-assumption-free complement to the linear model. Because several biomarkers correlated with BMI, partial Spearman correlations adjusting for BMI were also computed for the nutrient-biomarker pairs identified in the primary correlation analysis (Supplementary Table S2).

## 3 Results

### 3.1 Sample demographics and characteristics

The analytical sample had 351 participants: 80 men (22.8%) and 271 women (77.2%) (Table 1). Out of the men, 54 (67.5%) were in the young-old group (60–74 years) and 26 (32.5%) were in the old-old group (75–90 years). For women, 186 (68.6%) were young-old and 85 (31.4%) were old-old. The median age did not differ between men and women (men: 69 years, IQR 65–75; women: 70 years, IQR 66–76, *p* = 0.416). Women had a higher median BMI than men (30.0 vs. 27.7 kg/m^2^, *p* = 0.001), higher total cholesterol (5.5 vs. 5.0 mmol/L, *p* = 0.001), and lower creatinine (80.0 vs. 94.0 μmol/L, *p* < 0.001). Men had higher median sodium intake (2680.8 vs. 2264.4 mg/day, *p* = 0.047), as well as higher protein (52.1 vs. 46.8 g/day, *p* = 0.018), phosphorus (893.3 vs. 801.1 mg/day, *p* = 0.022), niacin (20.0 vs. 16.8 NE/day, *p* = 0.006), and iron (12.9 vs. 11.4 mg/day, *p* = 0.028). Glucose, urea, and triglyceride values did not differ between men and women (all *p* > 0.05).

**Table 1:** Baseline characteristics of study participants, stratified by sex (*n* = 351).

Variable	Men (*n* = 80)	Women (*n* = 271)	*p*-value	Test
Demographic				
Age (years), median (IQR)	69 (65–75)	70 (66–76)	0.416	MWU
Young-old (60–74 years), n (%)	54 (67.5)	186 (68.6)	0.850	χ^2^
Old-old (75–90 years), n (%)	26 (32.5)	85 (31.4)	—	
Anthropometry				
BMI (kg/m^2^), median	27.7	30.0	0.001	MWU
Overweight or obesity (BMI ≥ 25), n (%)	All ≥ 25 ^a^	All ≥ 25 ^a^	—	
Cardiometabolic biomarkers (median)				
Fasting glucose (mmol/L)	6.8	6.8	0.236	MWU
Total cholesterol (mmol/L)	5.0	5.5	0.001	MWU
Triglycerides (mmol/L)	1.65	1.70	0.616	MWU
Creatinine (μmol/L), median (mean)	94.0 (103.4)	80.0 (84.4)	< 0.001	MWU
Serum urea (mmol/L), median (IQR)	6.4 (5.1–7.8)	5.9 (4.7–7.6)	0.121	MWU
Biomarker prevalence				
Fasting glucose > 7.0 mmol/L, *n* (%)	34 (42.5)	105 (38.7)	0.520	χ^2^
Fasting glucose > 11.0 mmol/L, *n* (%)	13 (16.2)	28 (10.3%)	—	
Total cholesterol > 5.2 mmol/L, *n* (%)	31 (38.8)	162 (59.8)	0.001	χ^2^
Triglycerides > 1.7 mmol/L, *n* (%)	36 (45.0)	125 (46.1)	0.860	χ^2^
Selected nutrient intakes (median, per day) ^b^				
Protein (g)	52.1	46.8	0.018	MWU
Sodium (mg)	2680.8	2264.4	0.047	MWU
Phosphorus (mg)	893.3	801.1	0.022	MWU
Niacin (NE)	20.0	16.8	0.006	MWU
Iron (mg)	12.9	11.4	0.028	MWU
Socioeconomic profile				
Monthly income 100,000–200,000 KZT, *n* (%)	57 (71.2)	116 (42.8)	< 0.001	χ^2^
Monthly income < 100,000 KZT, *n* (%)	19 (23.8)	146 (53.9)	—	—
Urban residence, *n* (%)	56 (70.0)	202 (74.5)	0.507	χ^2^

IQR, interquartile range. BMI, body mass index. MWU, Mann–Whitney *U* test. χ^2^, chi-square test. KZT, Kazakhstani tenge. NE, niacin equivalents. ᵃ All participants met the inclusion criterion of BMI ≥ 25 kg/m^2^. ᵇ Nutrients showing a sex difference at *p* < 0.05 by Mann–Whitney U test; remaining intakes are in Table 2. Overall sample (*n* = 351): monthly income 100,000–200,000 KZT, 173 (49.3%); income below 100,000 KZT, 165 (47.0%); urban residence, 258 (73.5%).

Household income distribution differed by sex (χ^2^ = 22.5, *p* < 0.001): 71.2% of men reported a monthly household income between 100,000 and 200,000 Kazakhstani tenge (KZT), while 53.9% of women reported income below 100,000 KZT. Urban or rural residence did not differ by sex (70.0% of men and 74.5% of women lived in urban areas, *p* = 0.507).

### 3.2 Cardiometabolic biomarker profiles

Biomarker prevalence was high in both sexes and differed in several respects (Table 1). Among men, 42.5% had fasting glucose above 7.0 mmol/L, with 16.2% exceeding 11.0 mmol/L. Elevated total cholesterol (above 5.2 mmol/L) was present in 38.8% of men but in 59.8% of women, a difference of 21 percentage points (*p* = 0.001). Hypertriglyceridemia affected similar proportions of men (45.0%) and women (46.1%). Serum creatinine was higher in men (mean 103.4 μmol/L, median 94.0) than in women (mean 84.4 μmol/L, median 80.0, *p* < 0.001). Median urea was 6.4 mmol/L (IQR 5.1–7.8) in men and 5.9 mmol/L (IQR 4.7–7.6) in women (*p* = 0.121).

### 3.3 Micronutrient adequacy and macronutrient balance

Nutrient adequacy relative to the national reference values is presented in Table 2. Inadequacy affected more than 80% of participants in both sexes for potassium, calcium, vitamin C, vitamin E, and thiamine, with potassium and calcium inadequacy exceeding 97% in both sexes. Sodium showed the opposite pattern: fewer than 2% of participants in either sex fell below the reference value, and median intake reached 212.3% of the reference in men and 183.7% in women. Together with sodium, niacin was the only nutrient whose median intake exceeded the reference value in both sexes (125.2% in men, 119.8% in women). Protein intake covered about 74% of the reference value in both sexes, with more than half of participants below the 80% threshold. Women showed a higher proportion of the reference value than men for vitamin A, riboflavin, vitamin C, carbohydrates, and total energy (all *p* < 0.05). Men showed a higher proportion than women for sodium (*p* = 0.047). The remaining nutrients did not differ by sex (Table 2).

**Table 2:** Nutrient adequacy relative to the age- and sex-specific reference value.

Nutrient	Men median % reference value	Men % < 80% reference value ^a^	Women median % reference value	Women % < 80% reference value ^a^	*p* ^b^
Protein	74.4	57.5	74.6	59.0	0.989
Total fat	77.6	52.5	82.0	48.7	0.290
Carbohydrates	52.4	91.2	58.4	84.5	0.010
Dietary fiber	58.6	75.0	55.6	77.1	0.504
Sodium ^c^	212.3	1.2	183.7	1.5	0.047
Potassium	34.3	98.8	32.9	100.0	0.380
Calcium	33.6	97.5	38.1	97.4	0.266
Magnesium	86.9	42.5	88.5	38.7	0.137
Vitamin A (RE)	41.9	72.5	61.3	60.9	0.009
Vitamin E (TE)	43.7	91.2	38.9	91.1	0.170
Thiamine (B1)	48.1	85.0	49.5	89.7	0.874
Riboflavin (B2)	60.4	78.8	69.8	62.0	0.011
Niacin (NE)	125.2	20.0	119.8	12.2	0.801
Vitamin C	22.3	97.5	30.5	86.7	0.006
Energy (kcal)	64.2	80.0	68.4	69.7	0.035

RE, retinol equivalents. TE, tocopherol equivalents. NE, niacin equivalents. ᵃ Proportion of participants with intake below 80% of the reference value. ᵇ *p*-values from Mann–Whitney *U* tests comparing percentage of the reference value between sexes. ^c^ Sodium has no recommended daily allowance in the conventional sense. The value shown is expressed against the age-specific physiological requirement applied in this study (1,300 mg/day for ages 60–74 and 1,200 mg/day for ages 75–90), which is lower than, and not equivalent to, the WHO population intake limit of below 2000 mg/day (Section 2.2).

### 3.4 Sex-stratified correlations between nutrient intake and cardiometabolic markers

**Figure 1:**
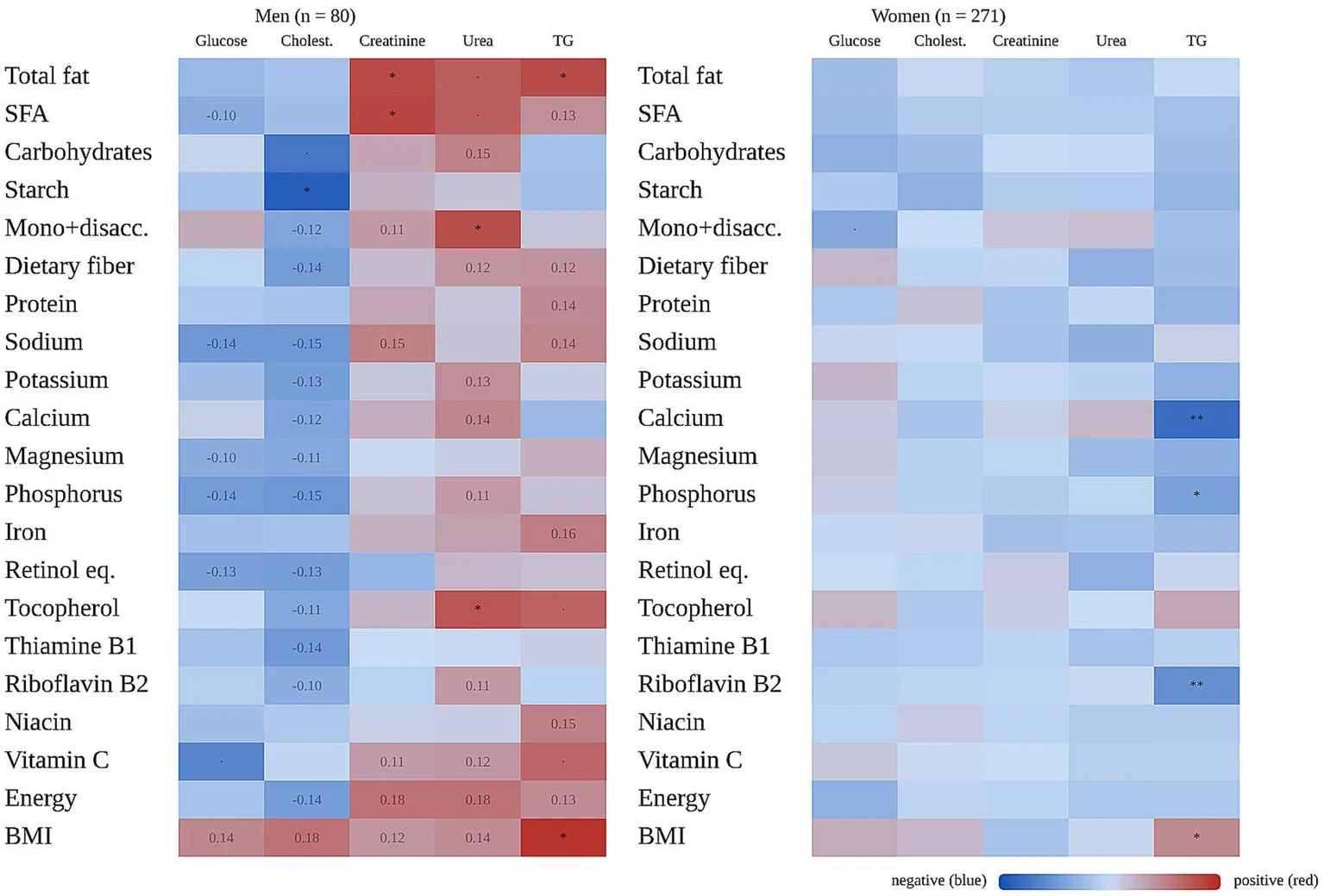
Spearman rank correlation coefficients between daily nutrient intake and cardiometabolic biomarkers, stratified by sex. Shading encodes direction and magnitude (red: positive; blue: inverse). Cells marked * *p* < 0.05; ** *p* < 0.01. Nutrients with |*rs*| < 0.10 and *p* ≥ 0.10 are shown without a value label.

Figure 1 presents Spearman rank correlations between daily nutrient intake and cardiometabolic biomarkers, stratified by sex. The pattern of associations differed between men and women.

In men, positive correlations were identified between total fat intake and serum creatinine (*r*s = 0.242, *p* = 0.031), between total fat and triglycerides (*r*s = 0.243, *p* = 0.030), and between SFA and creatinine (*r*s = 0.250, *p* = 0.025). Starch intake showed an inverse correlation with total cholesterol (*r*s = −0.269, *p* = 0.016). Mono- and disaccharide intake correlated positively with serum urea (*r*s = 0.237, *p* = 0.034), and tocopherol showed a positive association with urea (*r*s = 0.225, *p* = 0.045). BMI correlated positively with triglycerides in men (*r*s = 0.282, *p* = 0.012). Fasting glucose did not correlate with any dietary variable after sex stratification.

In women, most associations between nutrient intake and biomarkers were either absent or minimal. Dietary calcium showed a modest inverse correlation with serum triglycerides (*r*s = −0.237, *p* < 0.001), with weaker inverse correlations for riboflavin (*r*s = −0.164, *p* = 0.007) and phosphorus (*r*s = −0.126, *p* = 0.038). BMI had a weak positive correlation with triglycerides (*r*s = 0.139, *p* = 0.022). No other nutrient variable reached the conventional threshold for statistical reliability for any biomarker in the female group.

Because several biomarkers correlated with BMI, partial Spearman correlations adjusting for BMI were computed for the associations above (Supplementary Table S2). In men, the total fat–creatinine and SFA–creatinine correlations were largely unchanged after adjustment, while the fat–triglyceride correlation fell just past the conventional threshold (partial *r*s = 0.207, *p* = 0.069). In women, the calcium, riboflavin, and phosphorus correlations with triglycerides were not meaningfully changed by BMI adjustment.

### 3.5 Dietary fat intake and triglyceride concentration across intake tertiles

In men, total fat intake tertiles were defined as T1 (< 42.7 g/day), T2 (42.7–66.7 g/day), and T3 (> 66.7 g/day). Median triglyceride concentrations rose in step with total fat intake: T1 = 1.30 mmol/L, T2 = 1.60 mmol/L, T3 = 1.90 mmol/L, a 46.2% increase from the lowest to the highest tertile. The Kruskal-Wallis test did not reach the conventional threshold for statistical reliability (*p* = 0.068, approximately 27 men per tertile). No gradient emerged for glucose or cholesterol across total fat intake tertiles in men.

For women, total fat intake was categorized into tertiles as follows: T1 consisted of intakes below 41.9 g/day, T2 ranged from 41.9 to 60.3 g/day, and T3 exceeded 60.3 g/day. Median triglyceride levels were similar across these groups (T1 = 1.70, T2 = 1.70, T3 = 1.75 mmol/L, *p* = 0.726), with no dose-dependent relationship between total fat intake and triglyceride concentration in women. Carbohydrate intake tertiles showed no difference in fasting glucose for either sex (men: *p* = 0.955; women: *p* = 0.139), although median glucose was somewhat higher in the middle carbohydrate tertile among men (6.90 mmol/L) than in the lowest tertile (6.40 mmol/L).

### 3.6 Multiple linear regression of serum triglycerides

**Figure 2:**
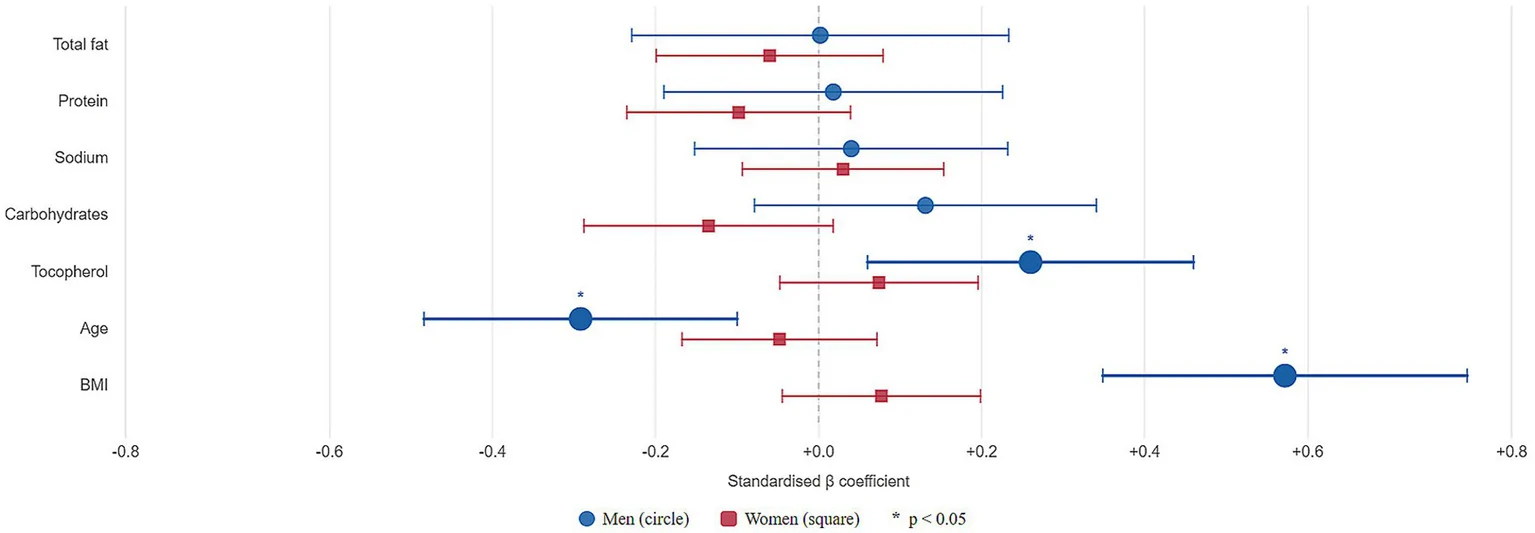
Standardized *β* coefficients (points) with approximate 95% confidence intervals (horizontal bars) from multiple linear regression of serum triglyceride concentration in men (circles) and women (squares). Predictors standardized to *z*-scores prior to analysis; *β* values are directly comparable across predictors and between sexes. Predictors reaching *p* < 0.05 are marked with *. Model *R2*: men = 0.333; women = 0.048.

A multiple linear regression analysis modeled serum triglyceride concentrations separately in men and women, with standardized predictors including total fat, carbohydrate, and protein intake, BMI, age, sodium, and tocopherol consumption (Figure 2). Results are summarized in Table 3. In men, the regression model accounted for about one-third of the variance in triglyceride levels (*R*^2^ = 0.333). Among predictors, BMI showed the strongest positive association (standardized *β* = 0.572), followed by a negative association with age (*β* = −0.292) and a positive association with tocopherol intake (*β* = 0.260). The contribution of dietary fat was minimal within this multivariate framework (*β* = 0.002), suggesting that its bivariate association with triglycerides was largely mediated by adiposity. The model explained a modest proportion of variance in women (*R*^2^ = 0.048), with no individual predictor exceeding a standardized effect of 0.15 in magnitude. Carbohydrate intake was the largest contributor in the female model (*β* = −0.135), followed by protein (*β* = −0.098) and BMI (*β* = 0.077), though none reached the conventional threshold for statistical reliability.

**Table 3:** Multiple linear regression of serum triglycerides on dietary and anthropometric predictors, stratified by sex.

Predictor	Men std. *β*	*p* (men)	Women std. *β*	*p* (women)
BMI (kg/m^2^)	0.572	< 0.01	0.077	NS
Age (years)	−0.292	< 0.05	−0.048	NS
Total fat (g/day)	0.002	NS	−0.060	NS
Carbohydrates (g/day)	0.131	NS	−0.135	NS
Protein (g/day)	0.018	NS	−0.098	NS
Sodium (mg/day)	0.040	NS	0.030	NS
Tocopherol (TE/day)	0.260	<0.05	0.074	NS
Model *R*^2^	0.333		0.048	

*β*, standardized regression coefficient. *R*^2^, coefficient of determination. NS, did not reach *p* < 0.05. TE, tocopherol equivalents.

### 3.7 Logistic regression analysis of hypertriglyceridemia risk

Hypertriglyceridemia, defined as serum triglyceride concentration above 1.7 mmol/L, was detected in 36 of 80 male participants (45.0%, Table 1) and 125 of 271 female participants (46.1%). Separate logistic regression models were built for each sex, using seven standardized predictors: total fat, carbohydrates, protein, BMI, age, sodium, and tocopherol. The resulting patterns showed sex-specific differences, detailed in Table 4.

**Table 4:** Sex-stratified logistic regression of hypertriglyceridemia (serum triglycerides > 1.7 mmol/L) on standardized dietary and anthropometric predictors (*n* = 351).

Predictor ^a^	Men (*n* = 79) *β* (SE); OR (95% CI); p	Women, base model ^b^ (*n* = 271) *β* (SE); OR (95% CI); *p*	Women, extended model (*n* = 271) *β* (SE); OR (95% CI); *p*
Total fat	−0.085 (0.384); 0.918 (0.43–1.95); 0.824	0.364 (0.188); 1.439 (1.00–2.08); 0.053	0.395 (0.191); 1.485 (1.02–2.16); 0.039
Carbohydrates	−0.653 (0.389); 0.520 (0.24–1.12); 0.093	−0.137 (0.154); 0.872 (0.64–1.18); 0.373	−0.129 (0.155); 0.879 (0.65–1.19); 0.405
Protein	−0.006 (0.441); 0.994 (0.42–2.36); 0.990	−0.438 (0.179); 0.645 (0.45–0.92); 0.015	−0.235 (0.210); 0.791 (0.52–1.19); 0.263
BMI	0.773 (0.311); 2.167 (1.18–3.99); 0.013	0.267 (0.133); 1.306 (1.01–1.70); 0.045	0.245 (0.135); 1.277 (0.98–1.66); 0.069
Age	−0.179 (0.268); 0.836 (0.49–1.41); 0.503	−0.111 (0.130); 0.895 (0.69–1.15); 0.391	−0.085 (0.131); 0.919 (0.71–1.19); 0.519
Sodium	0.061 (0.441); 1.063 (0.45–2.53); 0.890	0.138 (0.163); 1.148 (0.84–1.58); 0.396	0.148 (0.168); 1.160 (0.84–1.61); 0.376
Tocopherol (TE)	0.904 (0.461); 2.470 (1.00–6.10); 0.050	−0.032 (0.166); 0.968 (0.70–1.34); 0.846	−0.052 (0.176); 0.949 (0.67–1.34); 0.766
Calcium	n/a	n/a	−0.331 (0.187); 0.719 (0.50–1.04); 0.077
Riboflavin (B2)	n/a	n/a	−0.049 (0.196); 0.952 (0.65–1.40); 0.802
Model statistics			
AUROC ^c^	0.717	0.637	0.661
McFadden pseudo-R^2^	0.153	0.038	0.051
LR χ^2^ (p) ^c^	16.66 (p = 0.020)	14.38 (p = 0.045)	19.18 (*p* = 0.024)
Events / n	36/79	125/271	125/271

^a^ All predictors standardized to *z*-scores prior to analysis. ^b^ The base women’s model includes the same seven predictors as the men’s model; the extended model additionally incorporates dietary calcium and riboflavin. ^c^ AUROC, area under the receiver-operating characteristic curve. LR χ^2^, likelihood-ratio chi-square.

For the male cohort, the model showed moderate ability to discriminate cases (AUROC = 0.717, McFadden pseudo-*R*^2^ = 0.153, likelihood-ratio χ^2^ = 16.66, *p* = 0.020). Among the evaluated factors, BMI was the only predictor with an independent association with hypertriglyceridemia that reached the conventional threshold for statistical reliability (odds ratio = 2.17, 95% confidence interval 1.18 to 3.99, *p* = 0.013), consistent with its role in the linear regression. Tocopherol intake approached that threshold (odds ratio = 2.47, 95% confidence interval 1.00 to 6.10, *p* = 0.050). Carbohydrate intake showed a borderline inverse trend (OR = 0.52, 95% CI 0.24–1.12, *p* = 0.093).

The model for women showed lower predictive performance (AUROC = 0.637, McFadden pseudo-R^2^ = 0.038, likelihood-ratio χ^2^ = 14.38, *p* = 0.045). Protein intake was the only dietary factor with an independent inverse relationship to hypertriglyceridemia odds (OR = 0.64, 95% CI 0.45–0.92, *p* = 0.015). BMI retained a weak positive association (OR = 1.31, 95% CI 1.01–1.70, *p* = 0.045). Expanding the female model to include dietary calcium and riboflavin produced a modest improvement in fit (McFadden pseudo-*R*^2^ = 0.051, AUROC = 0.661). Within this extended model, total fat intake reached the conventional threshold for statistical reliability (OR = 1.49, 95% CI 1.02–2.16, *p* = 0.039), while calcium intake showed a borderline inverse association with hypertriglyceridemia (OR = 0.72, 95% CI 0.50–1.04, *p* = 0.077).

## 4 Discussion

In this sample of 351 older adults with confirmed alimentary-dependent diseases in Central Kazakhstan, reported energy and macronutrient intakes fell below the national reference values in both sexes, and sodium and niacin were the only nutrients whose median intake exceeded the reference. Against this background of low reported intake, potassium, calcium, vitamin C, vitamin E, and thiamine showed near-universal shortfalls. At the individual biological level, the associations that emerged in men, particularly between total fat intake and creatinine or triglycerides and the dominant role of BMI as a triglyceride predictor, had no real counterpart in women.

Potassium and calcium deficits were near-universal in this sample, with more than 97% of participants in both sexes consuming less than 80% of the recommended level for each. Low potassium intake matches the infrequent daily consumption of fruit, vegetables, and legumes documented in the dietary recall data, as these foods make up the primary sources of potassium in most diets. This pattern matches findings from other aging populations carrying multiple chronic conditions, although the degree of deficiency identified here appears particularly pronounced within that body of literature. This observation carries direct clinical weight, given that sustained potassium intake has shown measurable blood pressure-lowering effects in hypertensive individuals: several meta-analyses of randomized controlled trials have reported measurable reductions in systolic blood pressure following sustained increases in potassium intake (30, 31). The sodium intake measured here exceeded the national reference value by more than twofold in men and close to twofold in women, likely adding to vascular risk through the interaction between sodium and potassium in renal sodium reabsorption and vascular tone modulation (32).

The reference value applied to sodium in this analysis (1,300 mg/day for participants aged 60–74 years and 1,200 mg/day for those aged 75–90 years) reflects a physiological requirement level rather than the World Health Organization’s population-level ceiling of below 2000 mg/day. Expressed against that ceiling instead, the median intakes reported here (2680.8 mg/day in men and 2264.4 mg/day in women) equate to about 134 and 113%, respectively, a smaller excess than the reference-value framing suggests but an excess all the same. If anything, the sodium intake reported here is likely conservative relative to this population’s true habitual intake. A study using 24-h urinary sodium excretion, the reference method for sodium assessment, found a mean intake equivalent to 17.2 g of salt per day among Kazakhstani adults (33), and the World Health Organization has separately identified Kazakhstan as having one of the highest sodium intakes recorded worldwide (34). Self-reported 24-h recall is known to underestimate sodium intake relative to urinary excretion, since discretionary salt added during cooking and at the table is hard for respondents to quantify from memory (35). The gap between the recall-based estimate reported here and the urinary-based national figure fits this known pattern more closely than it fits a genuine improvement in sodium intake in this clinical population, although some participants may already have reduced intake following a hypertension diagnosis.

A separate, more recently described pathway may add to the sodium-blood pressure relationship above. High dietary sodium alters the composition of the gut microbiota, lowering populations of lactate-producing, blood-pressure-protective bacteria and shifting T-helper-cell balance toward a pro-inflammatory profile in animal models (36). Reviews of this pathway describe a route from high salt intake to increased intestinal permeability and systemic inflammation, which in turn interacts with the renin-angiotensin-aldosterone system to raise blood pressure (32, 37, 38). Whether this pathway operates in the present population cannot be tested with the data available here, but it offers a mechanism, beyond the direct renal sodium-potassium handling already described, through which the near-universal sodium excess and potassium shortfall observed in this sample could compound each other’s cardiovascular effect.

Median vitamin C intake met only 22.3% of the reference value in men and 30.5% in women, meaning most participants fall well short of levels needed to maintain adequate antioxidant function. Persistent low vitamin C status has been linked to impaired collagen synthesis, higher systemic inflammatory markers, and reduced cellular resilience against oxidative stress in older adults (13, 39). In people with T2DM, low vitamin C status has been associated with increased systemic inflammation and endothelial dysfunction (40). Similar patterns of inadequate vitamin C status have been reported in other older adult populations, suggesting that insufficient vitamin C intake remains a common nutritional concern across different care settings (41). This study extends those observations to people with established disease living in the community in Central Asia.

Vitamin E deficiency was widespread too within this sample: about 91% of both men and women did not reach 80% of the reference value. Tocopherol, as a lipid-soluble antioxidant in biological membranes and plasma lipoproteins, becomes depleted when oxidative stress rises. Vitamin E deficiency reduces antioxidant protection against lipid peroxidation and promotes oxidative modification of low-density lipoprotein, processes implicated in the development of atherosclerosis (42).

Thiamine shortfall, present in over 85% of both men and women, matters especially for people with T2DM. Thiamine pyrophosphate serves as an essential cofactor for pyruvate dehydrogenase and transketolase, thereby supporting mitochondrial energy metabolism and the pentose phosphate pathway (43). Without enough thiamine, glucose metabolism falters and oxidative by-products build up in active tissues, a process that can worsen cell damage linked to high blood sugar. The scale and breadth of these deficits point to the limits of food-based counseling as a sole strategy in this population and support the case for systematic screening of micronutrient status with prospective evaluation of supplementation approaches.

In men, dietary fat and SFA correlated with serum creatinine and triglycerides, starch showed an inverse relationship with total cholesterol, and mono- and disaccharide intake correlated with urea. In women, nearly all of these associations were absent. This sex divergence in dietary–biomarker correlations forms the central observational finding of the study. Given the exploratory nature of the correlation analysis and the absence of multiple comparison correction, these associations represent preliminary signals that call for replication with repeated dietary measurement before any causal inference can be drawn. The positive link between dietary fat and creatinine in men fits two mechanisms operating in parallel: greater skeletal muscle mass in men is associated with higher creatinine production, and fat-induced changes in glomerular hemodynamics, a consequence of increased dietary acid load, may mildly raise creatinine even without overt nephropathy (44). The positive association between dietary fat intake and triglycerides is biologically plausible because absorbed fatty acids contribute to hepatic triglyceride synthesis and very-low-density lipoprotein (VLDL) secretion, while long-term diets high in saturated fat have been associated with higher fasting triglyceride concentrations in both observational and intervention studies (45, 46).

In the multiple regression model for men, BMI rather than dietary fat emerged as the dominant triglyceride predictor, with dietary fat contributing minimally once adiposity was accounted for. This does not negate the dietary signal, it repositions it mechanistically. Excess visceral adiposity increases free fatty acid flux to the liver, promoting hepatic triglyceride synthesis and very-low-density lipoprotein (VLDL) secretion through mechanisms that extend beyond short-term variation in dietary fat intake (47, 48). The inverse age contribution fits evidence that hypertriglyceridemia peaks in middle age and declines in the very elderly as overall energy intake and appetite fall (49). The positive tocopherol coefficient should not be read as causal, and its association with triglycerides did not hold consistently once a log-transformed version of the outcome was tested as a sensitivity check. Tocopherol is fat-soluble and predominantly consumed in high-fat foods, so this association most likely reflects dietary fat co-variation rather than an independent tocopherol effect. The logistic regression analysis backed up the linear model findings. In men, BMI was the leading independent predictor of hypertriglyceridemia, with the model reaching moderate discriminative capacity. Tocopherol appeared as an additional correlate through the fat-intake collinearity mechanism noted above. In women, the same set of predictors performed considerably worse, with protein intake the only dietary variable showing an independent inverse association with hypertriglyceridemia odds. This association fits evidence that higher dietary protein relates to greater hepatic apolipoprotein B catabolism and reduced VLDL particle number (48), though the cross-sectional design cannot establish direction. In the extended women’s model incorporating calcium and riboflavin, total fat intake showed a modest positive association and calcium a borderline inverse association. The difference in model performance between sexes (Table 4) strengthens the point that standard dietary and anthropometric variables do not fully capture hypertriglyceridemia risk in women, suggesting unmeasured, sex-specific factors are at play.

The near-absence of dietary–biomarker associations in women raises questions about how well standard dietary-lipid models apply across sexes in this age group. At least two interpretive frameworks fit this pattern, although the cross-sectional design does not allow choosing between them. First, sex differences in postprandial lipemia are well documented. Following comparable dietary fat loads, women generally show a smaller postprandial rise in triglyceride concentrations than men, a pattern that has been linked to differences in lipoprotein lipase activity, skeletal muscle lipid oxidation, and body fat distribution (17, 50). Although the women in this sample were post-menopausal, persistent differences in body composition, including lower visceral adiposity and proportionally greater subcutaneous fat, may attenuate the dietary–lipid signal relative to men (15, 16, 51). Second, differences in body composition, adipose tissue distribution, and systemic inflammatory profiles between men and women may contribute to variability in circulating biomarker concentrations, potentially influencing the strength of observed dietary–biomarker associations (52). The present data fit both explanations, but neither can be confirmed without direct measurement of plasma volume, total body water, or circulating sex hormone concentrations, none of which were assessed in this study. The weaker BMI–triglyceride correlation observed in women relative to men aligns with prior reports of sex-related divergence in the adiposity–biomarker relationship in older cohorts (53).

The sex-specificity of the calcium and riboflavin associations with serum triglycerides in women has mechanistic grounding. Dietary calcium reduces intestinal fatty acid absorption through formation of insoluble calcium-fatty acid soaps in the gut lumen, limiting micellar solubilization of long-chain fatty acids (54). Calcium also modulates parathyroid hormone and calcitriol concentrations, and elevated parathyroid hormone secondary to calcium deficiency has been associated with impaired peripheral lipid clearance (55). In this population, where calcium intake reached only 33–38% of the reference value, even a modest increase in dietary calcium may produce a measurable effect on triglyceride homeostasis, given that dose–response relationships are typically steepest in the low-intake range.

The inverse correlation between riboflavin and serum triglycerides in women in this sample was not pre-specified and should be treated as a post-hoc, hypothesis-generating observation. Riboflavin serves as an essential cofactor for flavin-dependent enzymes involved in mitochondrial *β*-oxidation of fatty acids, and inadequate riboflavin status has been shown to impair mitochondrial energy metabolism and lipid oxidation (56). Whether this pathway operates in the present population cannot be determined from cross-sectional data, and unmeasured dietary confounders shared between riboflavin-rich foods and other lipid-modifying dietary components may contribute to the association. This finding calls for dedicated examination in prospective studies with repeated dietary assessment and objective biochemical measurement.

The dietary pattern outlined in this study carries particular weight for geriatric primary care in Kazakhstan and similar Central Asian regions. Widespread deficiencies in potassium and calcium, together with sodium intake approximately twice the recommended level, describe a nutritional profile that may raise blood pressure while limiting the diet’s ability to offset vascular risk. These three imbalances are modifiable through food selection, yet each was the rule rather than the exception in this community-dwelling sample. Primary care visits appear to be the most accessible point for intervention. A large share of participants in this sample named medical professionals as their primary source of dietary information, which places general practitioners in a position to influence dietary behavior at a meaningful scale.

The sex dimorphism identified here argues against applying uniform dietary counseling to men and women in this age group. For men, adiposity shows the strongest independent association with hypertriglyceridemia, and dietary fat correlates with both triglycerides and creatinine. Weight management and saturated fat reduction are therefore the most directly supported dietary priorities in this group. For women, in whom dietary–lipid associations were largely absent, dietary recommendations that increase low-fat dairy products, leafy greens, and riboflavin-rich foods such as eggs, liver, and lean meat address the micronutrient deficits most evident in this group. Age- and condition-specific dietary protocols for older adults are becoming more formalized in geriatric nutritional guidelines (8, 57), and this data supports the case for separate dietary advice for men and women in Central Asia.

Effective communication matters as much as the content of dietary advice. Complex nutrient prescriptions rarely translate into behavior change in populations with limited nutritional literacy. Plain-language guidance tied to familiar foods, such as encouraging daily inclusion of a dairy portion or fresh vegetables and a specific reduction in processed, salted products, outperforms detailed macronutrient goals in comparable age groups (58, 59). Systematic nutritional screening using the Geriatric Nutritional Risk Index or Mini Nutritional Assessment, followed by structured counseling, provides an evidence-based framework for chronic disease management in primary care geriatrics and health practice broadly (60–62). Building the capacity of both healthcare providers and patients to act on dietary information appears necessary given the low nutritional literacy documented in this sample.

Also, women in this cohort were more likely than men to report lower household income, a finding that appeared to reflect differences in household composition rather than sex alone. A substantially higher proportion of women were widowed and lived in smaller households, reducing the opportunity to pool pensions or other household income. Although socioeconomic variables were not included in the multivariable analyses, these findings highlight the potential influence of household structure on dietary resources and nutritional status among older adults.

Several limitations of this study call for explicit consideration. Most importantly, dietary intake was assessed by a single 24-h recall. A single recall is subject to substantial within-person day-to-day variability and does not reliably estimate habitual nutrient intake at the individual level. In nutritional epidemiology, this form of measurement error systematically pulls dietary–biomarker associations toward the null, meaning that true associations may be stronger than those detected here, while some observed null results may reflect measurement imprecision rather than genuine biological independence. Repeated, non-consecutive recalls, ideally spanning at least one weekday and one weekend day, materially improve the accuracy of usual-intake estimates relative to a single recall (35). Four to five recall days would have reduced, though not removed, this source of error in the present study. The exploratory correlation analyses are particularly sensitive to this source of error. Although the standardized CINDI protocol and the distribution of recalls across different weekdays reduce some systematic variance, these measures do not substitute for repeated dietary assessment. All dietary–biomarker associations reported here should be treated as hypothesis-generating.

Medication use was not systematically recorded and represents a material source of unmeasured confounding. Statins directly reduce circulating cholesterol and triglycerides; thiazide diuretics alter potassium and sodium handling; metformin and other hypoglycemic agents influence glucose and lipid metabolism. The degree to which pharmacological effects modified the observed dietary–biomarker associations cannot be determined from the available data. Whether participants with longer-standing T2DM had already received structured nutritional counseling was also not recorded. Prior counseling could have modified intake in ways that attenuate the associations reported here, particularly for sodium. Physical activity level, a major independent determinant of triglyceride concentration and insulin sensitivity, was not measured and could not enter any model.

Observed associations may also reflect reverse causation: individuals with established dyslipidemia or T2DM may already have changed their dietary habits in response to diagnosis. Biochemical data were extracted from routine ambulatory records rather than specimens collected under standardized research conditions, introducing potential variability in laboratory methods and pre-analytical handling. Laboratory timing was constrained to within 14 days of the dietary recall, but residual temporal mismatch between dietary exposure and biomarker measurement cannot be excluded. Triglyceride residuals from the linear regression departed from normality in both sexes. The binary logistic regression on hypertriglyceridemia was included as a distributional-assumption-free complement to the linear model, and the log-transformed sensitivity analysis (Supplementary Table S1) supports the stability of the main qualitative findings, though this combination does not fully resolve the issue of non-normally distributed continuous outcome data.

## 5 Conclusion

Older adults with co-occurring T2DM, arterial hypertension, and obesity in Central Kazakhstan consumed diets that fell well below recommended levels of potassium, calcium, vitamins C and E, and thiamine, while consistently exceeding sodium guidelines. These nutrient inadequacies followed a largely similar pattern across both sexes, suggesting that shared cultural and socioeconomic factors may shape their dietary environments.

The relationship between dietary intake and cardiometabolic biomarkers was far from symmetrical. In men, total fat and saturated fat intake showed positive correlations with creatinine and triglycerides, and adiposity was the dominant independent predictor of hypertriglyceridemia in both linear and logistic regression models. In women, those dietary–lipid associations were largely absent. Calcium and riboflavin emerged instead as inverse correlates of serum triglycerides, and dietary protein was the only independent predictor of hypertriglyceridemia in the logistic model. The logistic models performed better in men than in women, suggesting that standard dietary and anthropometric measures capture the biology of hypertriglyceridemia less completely in women. The pattern of dietary–biomarker associations identified here suggests that nutritional counseling for older adults with alimentary-dependent diseases should account for sex-related differences in metabolic physiology and nutritional priorities. Regardless of sex, both groups showed near-universal inadequacy in potassium, calcium, and antioxidant vitamins alongside sodium excess, deficits that are modifiable through food selection and that stand as priorities for primary care-based dietary intervention. Longitudinal studies incorporating repeated dietary assessment, standardized prospective biochemistry, and objective physical activity measurement are needed to determine the direction and clinical magnitude of the associations described here.

## Data Availability

The raw data supporting the conclusions of this article will be made available by the authors, without undue reservation.
