# Supplementary material for: Sex-specific associations between dietary nutrient intake and cardiometabolic biomarkers in older adults with diet-related chronic diseases
Source: Front Nutr. 2026 Aug 14;13:1877635. doi: 10.3389/fnut.2026.1877635 (PMC13521813; doi:10.3389/fnut.2026.1877635)
Supplement: Supplementary file 1 [file Table_1.docx]

Supplementary Material

# 1 Supplementary Tables

**Supplementary Table S1.** Sensitivity analysis using log-transformed serum triglycerides.

| **Predictor** | **Men β, raw (p)** | **Men β, log-TG (p)** | **Women β, raw (p)** | **Women β, log-TG (p)** |
| --- | --- | --- | --- | --- |
| BMI | 0.572 (< 0.01) | 0.211 (< 0.001) | 0.077 (0.395) | 0.050 (0.09) |
| Age | −0.292 (< 0.05) | −0.123 (< 0.05) | −0.048 (0.317) | −0.042 (0.16) |
| Model R² | 0.333 | 0.260 | 0.048 | 0.046 |

**Supplementary Table S2.** Zero-order versus BMI-adjusted partial Spearman correlations for nutrient-biomarker pairs.

| **Pair** | **Sex** | **n** | **Zero-order rs (p)** | **Partial rs adjusted for BMI (p)** |
| --- | --- | --- | --- | --- |
| Total fat – Creatinine | Men | 79 | 0.240 (0.033) | 0.226 (0.047) |
| SFA – Creatinine | Men | 79 | 0.254 (0.024) | 0.248 (0.029) |
| Total fat – Triglycerides | Men | 79 | 0.241 (0.033) | 0.207 (0.069) |
| Calcium – Triglycerides | Women | 271 | −0.237 (< 0.001) | −0.231 (< 0.001) |
| Riboflavin – Triglycerides | Women | 271 | −0.164 (0.007) | −0.159 (0.009) |
| Phosphorus – Triglycerides | Women | 271 | −0.126 (0.038) | −0.129 (0.034) |
